# Expression of Concern: Overexpression of Wnt7a enhances radiosensitivity of non-small-cell lung cancer via the Wnt/JNK pathway

**DOI:** 10.1242/bio.054924

**Published:** 2020-10-05

**Authors:** Pingping Ai, Xianhua Xu, Shijie Xu, Zhixia Wei, Shun Tan, Junzhe Li

The Editor-in-Chief of Biology Open has raised queries concerning the data shown in Biology Open (2020) 9, bio050575 (doi:10.1242/bio.050575)

The authors have been contacted for an explanation and have been asked to supply the original data.

Biology Open is publishing this note to make readers aware of the problem; we will provide further information once it has been resolved. This course of action follows the advice set out by COPE (Committee on Publication Ethics), of which Biology Open is a member.

